# Can bag-valve mask ventilation with positive end-expiratory pressure reduce hypoxia during intubation? A prospective, randomized, double-blind trial

**DOI:** 10.1186/s13063-021-05413-3

**Published:** 2021-07-17

**Authors:** Yili Dai, Jiayuan Dai, Joseph Harold Walline, Yangyang Fu, Huadong Zhu, Jun Xu, Xuezhong Yu

**Affiliations:** 1 Emergency Department, Peking Union Medical College Hospital, Chinese Academy of Medical Sciences and Peking Union Medical College, Beijing, China; 2 Accident and Emergency Medicine Academic Unit, Chinese University of Hong Kong, Prince of Wales Hospital, No. 30-32 Ngan Shing Street, Shatin, NT Hong Kong, China

**Keywords:** Bag-valve mask ventilation, Positive end-expiratory pressure, Hypoxia, Intubation

## Abstract

**Background:**

Hypoxia is one of the life-threatening complications of endotracheal intubation. Supplemental oxygen and ventilation play a vital role in preventing hypoxia. Bag-valve mask (BVM) ventilation is frequently used before intubation, and its ability to improve oxygenation was recently confirmed. It is still unclear if positive end-expiratory pressure (PEEP) added to BVM ventilation can further reduce hypoxia during intubation.

**Methods:**

This will be a prospective, randomized, double-blind trial to determine if PEEP combined with BVM ventilation can reduce the incidence of hypoxia during intubation compared with conventional BVM ventilation. The lowest oxygen saturation and incidence of complications will be compiled to verify the comparative effectiveness and safety of the two groups.

**Discussion:**

BMV ventilation with PEEP is hoped to further reduce the incidence of hypoxia during intubation.

**Trial registration:**

Chinese Clinical Trial Registry ChiCTR2000035156. Registered on August 2, 2020. It had begun enrollment after passing ethical review but before registration.

## Administrative information

**Table:** 

Title {1}	Can bag-valve mask ventilation with positive end-expiratory pressure reduce hypoxia during intubation? A prospective, randomized, double-blind trial
Trial registration {2a and 2b}.	This study has been registered with the Chinese clinical trial registry on August 2, 2020 (registration number ChiCTR2000035156).
Protocol version {3}	Version 1.0, 2020-08-02
Funding {4}	This study is funded by the CAMS (Chinese Academy of Medical Sciences) Innovation Fund for Medical Sciences (Project number 2017-I2M-1-009). The funding sponsored the design of the study and collection, analysis, and interpretation of data and in writing the manuscript.
Author details {5a}	Yili Dai, Jiayuan Dai, Yangyang Fu, Huadong Zhu, Jun Xu*, Xuezhong Yu* Emergency Department, Peking Union Medical College Hospital, Chinese Academy of Medical Sciences and Peking Union Medical College, Beijing, China. Joseph Harold Walline Accident and Emergency Medicine Academic Unit, Chinese University of Hong Kong, Prince of Wales Hospital, No. 30-32 Ngan Shing Street, Shatin, NT, Hong Kong, China Jun Xu and Xuezhong Yu are corresponding authors. Yili Dai, Jiayuan Dai and Joseph Walline are co-first authors. Jun Xu and Xuezhong Yu conceived and proposed the study. Yili Dai, Jiayuan Dai and Joseph Harold Walline was responsible for study design, statistical method selection and drafting the proposal. Yangyang Fu and Huadong Zhu took charge of modification and optimization of the scheme.
Name and contact information for the trial sponsor {5b}	Jun Xu, Peking Union Medical College Hospital 1 Shuaifuyuan, Dongcheng District, Beijing, China, Xujunfree@126.com Xuezhong Yu, Peking Union Medical College Hospital 1 Shuaifuyuan, Dongcheng District, Beijing, China, yxz@pumch.cn Yili Dai, Peking Union Medical College Hospital 1 Shuaifuyuan, Dongcheng District, Beijing, China, 2449771524@qq.com JiaYuan Dai, Peking Union Medical College Hospital 1 Shuaifuyuan, Dongcheng District, Beijing, China, 18311203788@163.com Joseph Harold Walline, Accident and Emergency Medicine Academic Unit, Chinese University of Hong Kong, Prince of Wales Hospital, No. 30-32 Ngan Shing Street, Shatin, NT, Hong Kong, China, jwalline@cuhk.edu.hk Yangyang Fu, Peking Union Medical College Hospital 1 Shuaifuyuan, Dongcheng District, Beijing, China, fuyangyang000@126.com Huadong Zhu, Peking Union Medical College Hospital 1 Shuaifuyuan, Dongcheng District, Beijing, China, zhuhuadong1970@126.com
Role of sponsor {5c}	The study sponsor and funders helped with the design of the study and collection, analysis, and interpretation of data and in writing the manuscript. They will have ultimate authority over any of these activities.

### Composition of the coordinating center and trial steering committee {5d}

The endpoint adjudication committee is composed of the heads of each hospital and will take counsel together to decide whether to terminate the study early.

## Introduction

### Background and rationale {6a}

Endotracheal intubation is a critical emergency medical procedure for patients who are unable to maintain adequate oxygen saturation or a patent airway. However, serious adverse events such as hypoxia, hypotension, aspiration, or cardiac arrest can occur during intubation. Among them, hypoxia is one of the most common complications in the emergency department (ED) [1–3]. This proportion is much higher than that seen in the operating room [4] because ED patients are most likely suffering from pathophysiological lung disease without the luxury of fasting or extensive pre-operative screening tests. Furthermore, hypoxia may reduce the first pass success of intubation [5] and associate with cardiac arrest [6]. It is therefore vital that we find better tools to reduce hypoxia during intubation in the ED.

A bag-valve mask (BVM) is a frequently used, portable, and economical ventilation device for improving patients’ oxygen saturation before intubation in the ED. The advantage of using BVM ventilation in different scenarios has been shown in multiple studies [7–9]. Additionally, a new randomized, controlled study of BVM ventilation demonstrated a decreased incidence of desaturation during intubation using BVM while not increasing the risk of aspiration or hypotension compared with no BVM ventilation [10, 11]. It remains unclear whether the effectiveness of BVM ventilation can be further improved, however. As a result, this trial will focus on the amelioration of BVM ventilation.

Applying a positive end-expiratory pressure (PEEP) valve to the BVM device allows for PEEP to be applied during BVM ventilations, potentially increasing the efficacy of BVM ventilation [12]. Before intubation, pre-oxygenation is routinely performed to improve a patient’s oxygen reserve and postpone the onset of hypoxia [13, 14]. Unfortunately, a high concentration of inhaled oxygen may also cause absorption atelectasis. The use of PEEP is one of the more effective ways to cope with atelectasis by preventing the collapse of the lung’s alveoli [15].

As we have already known, hypoxia commonly occurs and has a negative impact. BVM ventilation is widely used and shows few side effects. Although the utility of prophylactic BVM ventilation for patients with acute respiratory failure in the ICU has been demonstrated, few trials have assessed different BVM ventilation methods. This study will concentrate on the improvement of BVM ventilation to reduce hypoxia. It will verify whether adding PEEP to prophylactic BVM ventilation for ED patients during the intubation is superior to BVM alone in reducing hypoxia. This trial will be a randomized, double-blind, controlled trial to compare the effectiveness of BVM ventilation with or without PEEP. We hypothesize that BVM ventilation with PEEP may increase patients’ oxygen saturation and lower the risk of hypoxia.

### Explanation for the choice of comparators {6b}

Patients allocated to the control group will be ventilated using conventional BVM ventilation. All other patient characteristics and interventions will be the same as the intervention group.

## Objectives {7}

### Primary objective

To determine whether additional PEEP with BVM ventilation could improve the ventilation efficiency and lower the hypoxia compared with merely BVM ventilation in ED patients undergoing intubation. Hypoxia is defined as an oxygen saturation lower than 90%.

### Secondary objectives

The incidence of complications associated with BVM with or without PEEP, such as aspiration, hypotension, or cardiac arrest during intubation. Aspiration is defined as the presence of vomit seen in the glottic region during intubation. Hypotension is defined as a mean blood pressure lower than 65 mmHg.

### Trial design {8}

This will be a prospective, randomized, parallel group, controlled, double-blind, superiority trial to compare the effectiveness of BVM ventilation with or without PEEP to prevent hypoxia during intubation.

## Methods: participants, interventions, and outcomes

### Study setting {9}

This study will be led by the Emergency Department of Peking Union Medical College Hospital in Beijing, China. Three other hospitals will also enroll patients, including the First Affiliated Hospital of Xinjiang Medical University in the Xinjiang Uygur Autonomous Region, the Second People’s Hospital of Huai’an, and the Suqian People’s Hospital in Jiangsu province in China. These are all grade III A hospitals with adequate medical consultants, qualified doctors, and monitoring equipment to fulfill this study.

### Eligibility criteria {10}

#### Inclusion criteria

Patients requiring endotracheal intubation in the ED for acute respiratory failure will be recruited. Acute respiratory failure is defined as a respiratory rate higher than 30 breaths per minute, dyspnea at rest, a partial arterial oxygen pressure (PaO_2_) less than 60 mmHg on room air, or a PaO_2_ divided by the fraction of inspired oxygen (FiO_2_) less than 300 regardless of supplemental oxygen provided.

#### Exclusion criteria

Patients who are pregnant, less than 18 years old, or unwilling to participate in the study will be excluded. Patients with acute circulatory failure, cardiac arrest before intubation, anticipated difficult airway, chronic hypoxemia, or difficult BVM ventilation will be excluded as well.

Acute circulatory failure is defined as hypotension requiring vasopressor therapy to maintain a mean blood pressure more than 65 mmHg [16]. A difficult airway will be evaluated using the MACOCHA score, described in detail as Mallampati score III or IV, apnea syndrome (obstructive), cervical spine limitation, opening mouth less than 3 cm, coma, hypoxia, and anesthesiologist nontrained. A score less than 3 could almost exclude the difficult airway [17]. Difficult BVM ventilation means that it is not possible to provide adequate ventilation due to one or more of the following problems: inadequate seal, excessive gas leak, or excessive resistance to ingress or egress of gas [18].

#### Dropout criteria

Patients enrolled in this study quit for whatever reason before the ending or their data miss too much to analyze. Either situation could drop out the subjects.

Before the study, investigators will be trained to gather information timely and thoroughly explain the research process and related details to lower the possible dropouts.

## Interventions

### Intervention description {11a}

The settings for both groups are the same except for the extra PEEP. Oxygen flow rates will both be set at 15 l/min. The two-person, two-handed method will be used by two experienced ED providers in order to achieve an adequate seal [19, 20]. Ventilation will proceed at 12–20 breaths per minute until intubation. During this process, vital signs and other information will be recorded by staff trained in the research protocol.

### Criteria for discontinuing or modifying allocated interventions {11b}

Patients could opt out of the study at any time. If hypotension, reflux aspiration, or other possible adverse reactions occur during BVM ventilation, we will stop and take corresponding measures to treat any adverse reactions.

### Strategies to improve adherence to interventions {11c}

We hope to reduce any episodes of hypotension due to excessive lung expansion by controlling ventilation through appropriate monitoring. Gastric intubation and suction will be performed to reduce the incidence of aspiration, depending on fasting time.

### Relevant concomitant care permitted or prohibited during the trial {11d}

Non-invasive ventilation will not be used during the trial. Other airway interventions are permitted and will be noted on the study data form.

### Outcomes {12}

#### Primary outcome measure

The primary outcome measure is the lowest oxygen saturation recorded via pulse oxygen saturation monitoring during the intubation procedure. The intubation procedure starts when the laryngoscope blade is inserted and ends once the endotracheal tube position is confirmed. Since there may be further delays of the pulse oxygen saturation, data will continue to be recorded until 2 min after intubation ends.

#### Secondary outcome measures

The secondary outcomes deal with respiratory and circulatory complications. We will focus on two of these (severe hypoxia and aspiration) in terms of respiration. The number of patients whose pulse oxygen saturation is lower than 80% during the intubation procedure and the incidence of aspiration will be recorded. Aspiration is defined as the observation of stomach contents at the aditus glottidis by a laryngoscope during the intubation. As for circulation, we will pay close attention to the lowest systolic blood pressure reading, the number of patients with systolic arterial blood pressure lower than 90 mmHg, and any cases of cardiac arrest.

### Participant timeline {13}

**Fig. 1:**
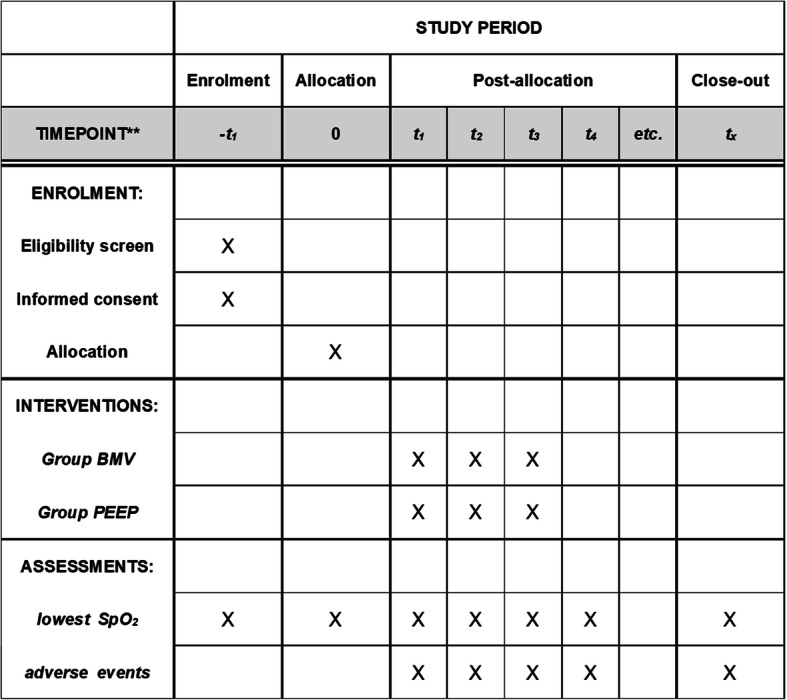
The schedule of enrolment, interventions, and assessments

The participant timeline is presented in Fig. 1.

### Sample size {14}

According to published studies, the mean oxygen saturation in control groups during intubation has been found to be 88% [21]. We posit that the intervention group may be increased by 5% and that the standard deviation will be 12%. Using a two-tailed α = 0.05, and β = 0.20, the sample size of the intervention group and control group were calculated using PASS 11 software to be N1 = N2 = 72 cases, or 144 cases total.

### Recruitment {15}

This study is expected to be carried out over 12 months. Patient recruitment and data collection will take place over a period of 10 months, from July 10, 2020, to May 10, 2021. An additional 2 months is being allocated for statistical analysis and write-up.

## Assignment of interventions: allocation

### Sequence generation {16a}

SPSS software will be used to generate the corresponding number of random sequences according to the sample size. And then, the random values will be divided equally into two groups as the control group and the intervention group. Patients involved in this research will be randomly assigned to these two groups. The grouping arrangement will not be changed once the research project is underway.

### Concealment mechanism {16b}

Sequentially numbered, opaque, sealed envelopes will be used to conceal the sequence until interventions are assigned.

### Implementation {16c}

Participating units will be trained to familiarize the study protocol, appoint a project manager, and obtain data collecting form in the launching conference. The manager in each unit will allocate the form to clinicians and supervise the research progress.

Patients will be selected based on their clinical characteristics and eligibility criteria by clinicians. After confirmation of inclusion, the clinician will contact the statisticians who generate the random sequence and access the grouping information. Then, they will assign the subjects to the intervention or control group accordingly and collect relative data.

## Assignment of interventions: blinding

### Who will be blinded {17a}

This will be a double-blind study. Neither patients, nurses, nor physicians performing BVM ventilation or intubation will know which BVM device will be used. To prevent any bias, identical-appearing BVM set-ups with either a 10-mm H2O of PEEP or a 0-mm H2O of PEEP valve will be used according to a patient’s group assignment.

### Procedure for unblinding if needed {17b}

Unblinding is not allowed unless multiple significant adverse events occur, and termination of the study is considered.

## Data collection and management

### Plans for assessment and collection of outcomes {18a}

Before randomization, some basic information will be recorded: patient identification number, age, sex, height, weight, body mass index, primary diagnosis, reason for intubation, systolic blood pressure, vasopressor use before intubation including the type and dosage, and any oxygen therapies used, including FiO_2_ and duration of use, pulse oximetry (SpO_2_) before intubation, and recent blood gas analysis (pH, PaO_2_, arterial carbon dioxide partial pressure (PaCO_2_)); any history of a difficult airway, airway evaluation using the MACOCHA score, and the time since the patient’s last meal will be recorded.

According to this basic information, patients who meet eligibility criteria will be randomized. A member of the research staff will select the corresponding BVM device for use prior to intubation. During the procedure, another member of the research staff will record key data. For example, the induction medicine (including drug name and dosage), the duration of BVM ventilation, the SpO_2_ before inserting the laryngoscope, the duration between inserting the laryngoscope and 2 min after intubation, the number of intubation attempts, the lowest SpO_2_ during the procedure, the lowest systolic blood pressure, any vasopressor use, and any incidence of aspiration or cardiac arrest.

During prophylactic BVM ventilation, some additional parameters will be monitored. Data such as exhaled tidal volume, respiratory rate, exhaled oxygen concentration, and partial pressure of end-tidal carbon dioxide will be collected to guarantee consistent manipulation and avoid bias caused by improper operation.

Intubation will be performed by physicians with at least 3 years of previous intubation experience to ensure patient safety. All data will be recorded in the Case Record Form and managed by the ResMan clinical trial public management platform. After collection, data will be analyzed by the statistician in our research group.

### Plans to promote participant retention and complete follow-up {18b}

Data collection and recording will be carried out by specific research study staff to ensure data integrity. Some data will also be checked using the memory of patients’ monitors.

### Data management {19}

Data will be collected on a Case Record Form (CRF) table and managed on the ResMan clinical trial public management platform (http://www.medresman.org.cn). Two investigators will be responsible for data entry and verification in each enrolling hospital. Research progress will be checked regularly.

## Statistical methods

### Statistical methods for primary and secondary outcomes {20a}

Data processing will be performed by the statistician in our research group and analyzed under an intention-to-treat premise. A two-tailed p-value less than 0.05 will be defined as statistically significant.

#### Description of patients’ basic characteristics

Measurement data will be described using means and standard deviations or median and inter-quartile range based on the data distribution characteristics. Calculated data is displayed as a frequency or a percentage.

#### Analysis of the primary outcome

The lowest SpO_2_ values of each subject will be imported to the SPSS software. Then, the normal distribution test and homogeneity test of variance will be performed. Finally, according to the results, the lowest SpO_2_ will be compared by the independent sample T-test or rank-sum test.

#### Analysis of secondary outcomes

The frequency and incidence rate of relevant complications, including SpO_2_ less than 80%, aspiration, systolic arterial blood pressure less than 90 mmHg, cardiac arrest, will be calculated in both groups. Then, based on the number of complications, select the chi-square test or Fisher’s exact probability test for comparison.

### Methods for additional analyses (e.g., subgroup analyses) {20b}

No subgroup analyses will be performed.

### Methods in analysis to handle protocol non-adherence and any statistical methods to handle missing data {20c}

This study adopts an intention-to-treat strategy to conduct data analysis. Subjects will be excluded if significant outcome data is missing.

## Oversight and monitoring

### Composition of the data monitoring committee, its role, and reporting structure {21a}

Composition of the data monitoring committee (DMC) will be made up of one specific person from each enrolling hospital. They will supervise study execution and data entry. The DMC member from Peking Union Medical College Hospital will be responsible for keeping the project on schedule. The DMC is independent from the sponsor and any competing interests.

### Interim analyses {21b}

If the intervention group shows any clear harms to patients (e.g., a statistically higher incidence of complications), the study will be terminated early. A check for elevated risk will occur at 6 months by the endpoint adjudication committee (see below).

### Adverse event reporting and harms {22}

If the intervention group shows any clear harms to patients (e.g., a statistically higher incidence of complications), the study will be terminated early. A check for elevated risk will occur at 6 months.

### Frequency and plans for auditing trial conduct {23}

The auditing team is composed of the ethics committee or related department members in each hospital. Furthermore, they will conduct the auditing monthly to track the study progress and data records. This process will be independent of the investigators or the trial sponsor.

### Plans for communicating important protocol amendments to relevant parties (e.g., trial participants, ethical committees) {25}

During the study, investigators carry out data collection in strict accordance with the protocol. Any questions to the protocol will be consulted with the head of their hospital. If involving scheme modification, it needs to be decided by collective consultation in all units. Finally, the updated scheme will be distributed to various research units and submitted to their respective ethical committees.

### Who will take informed consent? {26a}

Patients who meet the eligibility criteria will be provided with informed consent by the investigator. Their next of kin can decide whether to participate in this study on the patient’s behalf. After informed consent is given, they will be allocated to different groups according to the randomization sequence.

### Additional consent provisions for collection and use of participant data and biological specimens {26b}

This study does not require additional collection of patients’ blood samples or other specimens.

### Confidentiality {27}

Patients’ medical records (study records/CRF, laboratory tests, etc.) will be fully preserved at the hospital where the patients were enrolled. Researchers will be allowed access to their medical records. All personal information, including name, phone number, email address, etc. will not appear in the electronic database. Any public report of the results of this study will not disclose patients’ personal identity or information.

### Competing interests {28}

The authors declare that they have no competing interests.

### Provisions for post-trial care {30}

This trial will not require additional laboratory tests, and the relevant monitoring data will be free. In addition, participants who have adverse events on account of the trial will be provided timely treatment and reasonable financial compensation by the research group.

### Dissemination plans {31a}

Trial results will be communicated to others via publication in high-quality international medical journals.

### Plans to give access to the full protocol, participant-level data, and statistical code {31c}

The datasets used and/or analyzed during the current study will be available from the corresponding author(s) on reasonable request. Contact the corresponding author(s) through the email with valid reasons, and they will send the data accordingly.

### Plans for collection, laboratory evaluation, and storage of biological specimens for genetic or molecular analysis in this trial/future use {33}

This study does not require any additional collection of patients’ blood samples or other specimens.

## Discussion

Given the high incidence of hypoxia among ED patients requiring intubation, and the as-yet unclear need for PEEP during BVM ventilation, we designed this randomized, controlled trial to determine if there is any benefit to providing PEEP during BVM ventilation prior to intubation.

Inhalation oxygen concentration and PEEP are two important factors for maintaining oxygenation in intubated patients. These two factors may play a vital role in patients who are about to be intubated as well. Additional PEEP may increase the oxygen reserve and is expected to further enhance oxygenation in patients with hypoxia.

In this trial, we select patients with mild hypoxia, which may not cause patient-oriented harm. However, the additional attention paid to the saturation improvement during intubation may prevent a worse situation at an early stage. Therefore, it may not have a considerable impact but will never be indifferent.

## Trial status

Protocol version 1.0 has been registered on 2020-08-02. Patient recruitment and data collection will take place over the course of 10 months, from July 10, 2020, to May 10, 2021.

## Data Availability

The datasets used and/or analyzed during the current study are available from the corresponding author(s) on reasonable request. Contact the corresponding author(s) through the email with valid reasons, and they will send the data accordingly.

## Abbreviations

BVM: Bag-valve mask ventilation
PEEP: Positive end-expiratory pressure
ED: Emergency department
PaO_2_: Partial arterial oxygen pressure
FiO_2_: Fraction of inspired oxygen
PaCO_2_: Arterial carbon dioxide partial pressure
